# The 24-h Movement Compositions in Weekday, Weekend Day or Four-Day Periods Differentially Associate with Fundamental Movement Skills

**DOI:** 10.3390/children8100828

**Published:** 2021-09-22

**Authors:** Clare M. P. Roscoe, Michael J. Duncan, Cain C. T. Clark

**Affiliations:** 1 Human Sciences Research Centre, University of Derby, Derby DE22 1GB, UK; 2 Centre for Sport, Exercise and Life Sciences, Coventry University, Coventry CV1 5RW, UK; michael.duncan@coventry.ac.uk; 3 Centre for Intelligent Healthcare, Coventry University, Coventry CV1 5RW, UK; ad0183@coventry.ac.uk

**Keywords:** physical activity, fundamental movement skills, compositional data analysis, preschool children

## Abstract

The aim of this study was to investigate the relationship between weekday, weekend day and four-day physical activity (PA) behaviours and fundamental movement skills (FMS) in British preschool children from a low socio-economic status background using compositional data analysis (CoDA). One hundred and eighty-five preschool children aged 3–4 years provided objectively assessed PA and sedentary behaviour (SB) data (GENEActiv accelerometer) and FMS (TGMD-2). The association of 24-h movement behaviours with FMS was explored using CoDA and isotemporal substitution (R Core Team, 3.6.1). When data were considered compositionally (SB, light PA (LPA), moderate and vigorous PA (MVPA)) and adjusted for age, BMI and sex, the weekday-derived composition predicted total motor competence (r^2^ = 0.07), locomotor (r^2^ = 0.08) and object control skills (r^2^ = 0.09); the weekend day-derived composition predicted total motor competence (r^2^ = 0.03) and object control skills (r^2^ = 0.03), the 4-day-derived composition predicted total motor competence (r^2^ = 0.07), locomotor (r^2^ = 0.07) and object control skills (r^2^ = 0.06) (all *p* < 0.05). Reallocation of 5 min of LPA at the expense of any behaviour was associated with significant improvements in total motor competence, locomotor and object control skills; for weekend-derived behaviours, MVPA was preferential. Considering movement behaviours over different time periods is required to better understand the effect of the 24-h movement composition on FMS in preschool children.

## 1. Introduction

Physical activity (PA) during the preschool years is critical to a child’s development, overall health and well-being, concomitant to reducing the likelihood of disease and illness during and beyond childhood [1,2,3]. The benefits of PA and low exposure to sedentary behaviours (SB) in childhood are evidenced across empirical investigations [4]. It has been recommended that preschool children in the UK should be participating in at least 180 min of PA per day and this should include 60 min of moderate to vigorous PA (MVPA) [5]. Unfortunately, preschool children spend the majority of their day in SB and a low proportion of their day in MVPA (<15%) [6,7,8,9,10,11,12] with only one in 10 children meeting the recommendations of at least 180 min of PA per day in England [13].

Preschool children not adhering to the recommended PA guidelines represent a concern, especially those from a lower socio-economic status (SES) background, who have less access to PA opportunities and less chance for the development of fundamental movement skills (FMS) [14]. Fundamental movement skills are commonly developed in early childhood and provide the building blocks for future motor skills and lifelong PA [14,15,16]. Preschool children with better-developed FMS tend to spend significantly more time engaged in MVPA and significantly less time sedentary, compared to those with less developed FMS [17,18]. Improving preschool children’s FMS competency may facilitate engagement in higher levels of PA [19] and aid in reducing the risk of being overweight or obese [20].

It is of interest to determine how preschool children’s PA is accrued during the weekdays, when they attend preschool, in the weekend days, when they are influenced by their home environment and when combining weekend and weekdays. Indeed, weekday vs. weekend PA levels have routinely been reported to vary [19,21,22,23], with studies from Australia and England suggesting that preschool children are more physically active on weekend days [23,24], and others reporting greater MVPA engagement during the weekdays in Sweden, Denmark, England and Finland [11,19,22,25,26]. Therefore, with these discrepancies, vital additional research is required to identify any differences in PA levels and SB between weekdays, weekend days and four-day combinations in preschool children, considering the influence of FMS concurrently. This would help to gain imperative data to inform future public health interventions for preschool children. With this in mind, recently, compositional data analysis (CoDA) has taken precedence in the assessment of FMS and PA associations’ largely driven by the notion that PA behaviours over 24 h are time-use data and that considering such behaviours independently is statistically incongruent and may yield spurious inferences [27]. However, despite the adoption of a CoDA paradigm, the impact of combining data from different weekday and/or weekend days on the overall movement composition has not yet been discerned. Therefore, it remains unknown how time reallocated (from SB, light PA (LPA) and MVPA), using isotemporal modelling, might affect FMS scores in this population. Understanding preschool children’s movement behaviours and if these behaviours influence their FMS is particularly important, as this approach could prove vital in guiding public health interventions.

To the best of our knowledge, no study has considered a CoDA approach to estimate FMS when fixed durations of time for different movement behaviours have been reallocated during weekdays, weekend days and four days; this information is essential for guiding future public health interventions. Therefore, this present study sought to investigate the relationship between weekday, weekend day and four-day behaviours and FMS in British preschool children from a low SES using CoDA.

## 2. Materials and Methods

### 2.1. Participants and Data Collection

Following institutional ethics approval from the host University (P45654) and informed consent, children from 11 preschools in North Warwickshire, England, participated in this study. Data was collected between April 2014 and January 2017. Children’s assent was gained through their desire to be involved in the testing, and those unwilling to participate were removed from the study. The participants were a convenience sample and included 185 preschool children (99 boys, 86 girls), aged 3–4 years. North Warwickshire was chosen as it incorporates preschools that are considered to have the highest levels of deprivation in the County [28].

### 2.2. Anthropometric Assessment

Height was measured, to the nearest millimetre, using a portable stadiometer (Leicester Height Measure, Leicester, UK). Body mass was measured, to the nearest 0.1 kg, using portable weighing scales (Tanita BF350, Tokyo, Japan); the children were lightly dressed (t-shirt and light trousers/skirt) and barefoot or in socks. BMI was calculated as kilograms per square of the height in meters [29]. BMI was compared to a BMI-for-age chart to determine whether the child was of normal weight or overweight (≥95th percentile); this is recommended as a reasonable measure for assessing if children are overweight [30,31,32,33]. Waist circumference (WC) was measured, to the nearest centimetre, midway between the lowest rib and the iliac crest [34], using a non-elastic flexible tape while the child was in a standing position. WC was compared to standardised international cut-off points, and weight status was categorised as underweight, normal weight or overweight/obese [35].

### 2.3. Assessment of Physical Activity

PA was measured using a GENEActiv waveform triaxial accelerometer (ActivInsights Ltd., Cambridge, UK). The accelerometer recorded at a sampling frequency of 100 Hz [36,37,38]. The accelerometer was attached to an arm using a watch strap and positioned over the dorsal aspect of the dominant wrist, midway between the radial and ulnar styloid process. The participants wore the accelerometers for four consecutive days; this included two week and two weekend days [29]. Non-wear time was defined as 90 min windows of consecutive zero or nonzero counts [39]. The amount of wear time and percentage (%) of wear time that each child spent performing PA at different intensities were calculated for weekdays and weekend days. Measuring PA over 4 days, including one weekend day, is considered acceptable [29]. Due to the age of the participants and the difficulty for children to wear accelerometers for sustained periods of time, children were included in the final data analysis if the accelerometer had been worn for three days, including one weekend day and for a minimum of 6 h each day [29,40,41]. Accelerometer data were recorded for 178 of the 185 children; data from 7 children were not useable due to either the children not wearing the accelerometers or technical difficulties with them. For every epoch (number of seconds), movement data (activity counts) were added, logged, processed and analysed. Accumulated activity counts were categorised in terms of intensity: SB, LPA and MVPA [1]. The following cut points for 3–4-year-olds were used to determine PA intensity: dominant hand <8.1 cpm for SB, 8.1–9.3 cpm for LPA and 9.3+ cpm for MVPA [41]. For the non-dominant hand, <5.3 cpm for SB, 5.3–8.6 cpm for LPA and 8.6+ cpm for MVPA [41]. Using the GENEActiv post-processing software, the raw 100 Hz triaxial GENEActiv data were summed into a signal vector magnitude and expressed in 10 s epochs [42]. Children were classified as either meeting (sufficiently active) or not meeting (insufficiently active) the UK recommended 180 min a day of PA for 0–5-year-olds [5].

### 2.4. Assessment of FMS

An adapted version of the Test of Gross Motor Development2 (TGMD-2) was employed as a measure of FMS [43], with the removal of the underhand roll and the addition of skipping. Skipping was included because it benefits children’s physical fitness, improves their balance and muscle coordination, whilst being an enjoyable low-cost activity [44,45]. The underhand roll was removed, as a throwing activity (overhand throw) was already present, and the researchers introduced an assessment for balance to evaluate the all-round FMS skills of the children. TGMD-2 is a process-orientated test that examines a subset of locomotor and object control skills [46,47]. The unmodified TGMD-2 has been described as having an established validity and reliability amongst preschool children, with a test–retest reliability of 0.82–0.95 [23,47]. Prior to data collection, a senior member of the research team with experience of delivering the TGMD-2 protocol trained the field tester (primary researcher). The TGMD-2 was used in this study due to its availability and because collection of normative data was still occurring between 2014 and 2017 for the TGMD-3 [48]. The children were assessed in small groups (2–3), and the tests were administered by one tester to ensure consistency. The tests took part in an outside area or an adjacent primary school’s hall. The skills were physically demonstrated and verbally explained to ensure all children had the same information. If any child did not understand a task, then they were provided with a further verbal description and asked to repeat the trial of the skill again [49]. All children had a practice attempt prior to being scored on their two tests. All children were videoed completing the skills, using a camcorder (Sony, Tokyo, Japan) at standard frame rate, allowing the skills to be analysed after the occasion. All 12 skills were assessed in a standardised order, and the testing took between 30 and 35 min per group. The skills were performed in the following order: run, gallop, hop, leap, horizontal jump, skip, slide (locomotor skills), followed by two-handed strike of a stationary ball, stationary bounce of a ball, catch, kick and overhand throw (object control skills) [46,47]. The children’s FMS competency was assessed using the guidelines of the TGMD-2 protocol [43]. All video analyses were completed by the field tester. Intertester reliability was established prior to the commencement of testing using pre-coded videos of 10 children. There was 84.5% agreement across the 12 skills (range = 81.7–88.4%); this was similar to work by Foulkes et al. (2015) [49]. Intratester reliability was also determined using pre-coded videos of an additional 10 children, with the test–retest completed 1 week apart. In this case, 93.9% agreement was determined across the 12 skills (range = 90–98%); an 80–85% level of percentage agreement has been deemed acceptable [50]. For both trials of the skills, run, gallop, hop, jump, slide, strike, catch, kick and throw were scored out of 4, and leap, skip and bounce were scored out of 3. The scores were totalled over two attempts to provide the locomotor, object control and total gross motor skill score for a child (total FMS) [46,47].

### 2.5. Data Analysis

Compositional and isotemporal data analyses were conducted in R (http://cran.r-project.org, accessed on the 28 March 2020) using the compositions (version 1.40-1) [51], robCompositions (version 0.92-7) [52], and lmtest (version 0.9-35) packages, respectively. The daily composition (daily time spent in SB, LPA, MVPA) was defined in terms of central tendency, that is, the geometric mean of time spent in each component, linearly adjusted so that all components summed to the total daily behavior for interpretation in minutes per day, which for the purposes of this study, was bound to 600 min (10 h). Multivariate dispersion of week-, weekend- and 4-day-derived movement compositions were then described using pairwise log-ratio variation [53,54].

Multiple linear regression was used to investigate the relationship between week-, weekend- and 4-day-derived movement compositions (explanatory variable) and FMS and its subsets (dependent variable). Prior to inclusion in the regression model, the composition was expressed as a set of two isometric log ratio (*ilr*) co-ordinates. Covariates (age, BMI, sex) were also included. The *ilr* multiple linear regression models were further inspected for linearity, normality, homoscedasticity and outlying observations to ensure the assumptions were not violated. The significance of the week-, weekend- and 4-day-derived activity compositions (i.e., the set of *ilr* coordinates) was examined with the ‘car::Anova()’ function, which utilizes the Wald Chi squared statistic to calculate Type II tests, according to the principle of marginality, testing each covariate after all others [55].

The above *ilr* multiple linear regression models were used to predict differences in motor competence (and its subsets) associated with the reallocation of a fixed duration of time between two activity behaviours, which in this study, was set at 5 min, whilst the others remain unchanged. This was achieved by systematically creating a range of new activity compositions to mimic the reallocation of 5 min between all activity behavior pairs, using the mean composition of the sample as the baseline composition. The new compositions were expressed as *ilr* coordinate sets, and each was subtracted from the mean composition *ilr* coordinates to produce *ilr* differences. These *ilr* differences (each representing a 5 min reallocation between two behaviours) were used in the linear models to estimate differences (95% CI) in all outcomes (motor competence, locomotor score, object control score). The decision was made to only use 5 min reallocation, so to reflect the potential for real or actual change in MVPA and to exemplify the usefulness isotemporal substitution and compositional perspectives can yield. Furthermore, substituting too high a proportion of MVPA would render inferences spurious.

## 3. Results

### 3.1. Descriptive Statistics

Compositions and associated minutes per day for SB, LPA and MVPA, for week, weekend and 4-day derived behaviours, respectively, are presented in Table 1.

The variability of the week, weekend and 4-day behavior (SB/LPA/MVPA) compositions, respectively, is summarised in the variation matrix (Table 2) containing all pair-wise log-ratio variances. A value close to zero implies that the time spent in the two respective behaviours is highly proportional. For instance, the variance of log (Sedentary/MVPA) is 0.22 for week-derived behaviours, which reflects the (proportional) co-dependent relationship between the two behaviours. The highest log-ratio variance identified across the week-, weekend- and 4-day-derived composition was found between LPA and MVPA in the weekend derivations (0.99), suggesting that time spent in MVPA is the least co-dependent on LPA on the weekend.

### 3.2. Linear Regression

Data were initially examined using linear regression for each movement behaviour, independently. Results highlighted that only LPA significantly predicted total motor competence and the locomotor and object control subsets (Table 3).

### 3.3. Compositional Analysis and Isotemporal Substitution

When data were considered compositionally, adjusted for age, BMI and sex, their week-derived composition significantly predicted total motor competence (*p* = 0.007; r^2^ = 0.07), locomotor skills (*p* = 0.001; r^2^ = 0.08) and object control skills (*p* = 0.001; r^2^ = 0.09); their weekend-derived composition significantly predicted total motor competence (*p* = 0.04; r^2^ = 0.03) and object control skills (*p* = 0.04; r^2^ = 0.03) but did not significantly predict locomotor skills (*p* = 0.09; r^2^ = 0.01); their 4-day-derived composition significantly predicted total motor competence (*p* = 0.001; r^2^ = 0.07), locomotor skills (*p* = 0.002; r^2^ = 0.07) and object control skills (*p* = 0.005; r^2^ = 0.06).

Subsequent isotemporal substitution discerned that, based on the 95% CIs, for week- and 4-day-derived behaviours, adding LPA at the expense of any behavior was associated with significant improvements in total motor competence and the locomotor and object control subsets. However, for weekend-derived behaviours, MVPA was preferential to LPA, whilst the association between reallocating time for MVPA and SB was equivocal for week-, weekend-, and 4-day-derived behaviours, respectively (Table 4).

## 4. Discussion

To our knowledge, no study has adopted a CoDA approach to differentially estimate FMS when fixed durations of time for movement behaviours have been reallocated during weekdays, weekend days and 4-day behaviours in preschool children. Therefore, we aimed to investigate the relationship between weekday, weekend day and 4-day behaviours and FMS in British preschool children from a low SES background. A wealth of prior studies have examined the relationship between PA and FMS [56,57], SB and FMS [17] and between 24-h compositional movement behaviours and FMS. This study, however, analysed children from low-socioeconomic status backgrounds and addressed how week, weekend, and four-day movement behaviours associate with FMS, utilizing CoDA and isotemporal substitution.

Preschool children in this study reported on average 40 min/day of light and MVPA during weekdays and 13 min/day during weekend days, along with low FMS scores for age and sex. It is inappropriate to consider each movement behavior in an isolated manner; rather, a composition of daily behaviour should be determined, because 24-h movement is time-use data and bound to 1440 min a day [27,58]. Indeed, 24-h movement behaviours affect and are affected by all other behaviours during the day, as they co-exist in the same temporal paradigm. [59]. The results from the current study show that when applying CoDA, British preschool children responded in a significantly positive manner to adding LPA and removing SB during week, weekend, and 4 days. Ensuring LPA is participated in as opposed to SB is beneficial to children’s health [60]. Global PA guidelines encourage preschool children to be less sedentary and, ideally, participate in more MVPA for their physical health, mental health and well-being [61,62]. In the present study, we noted that adding LPA, at the expense of SB, was significantly associated with improvements in total motor competence and in the locomotor and object control subsets. Additionally, replacing LPA with MVPA in weekends suggested that positively greater FMS scores for locomotor, object control and total FMS may be gained. The opposite happened in weekdays, as replacing LPA with MVPA negatively impacted FMS. One possible explanation is that LPA during weekdays could be representative of a manipulative type of activities in the preschool setting which contributes to FMS, as the children were more active during the weekdays; however, in weekends when MVPA appeared to be much lower, an increase in MVPA is also needed for FMS improvement. We are therefore suggesting that the type of activities the children participate in are impactful on FMS. Though this is purely a conjecture, yet it does highlight the need to better understand not just the intensities of PA, but also “what” actual activities are performed. Without deeper insight into the specific activities being performed, we can only speculate that certain activities are more beneficial for skill development than others. Interestingly, we found that the replacement of SB with MVPA did not appear to uniformly elicit significantly positive changes across the week, weekend days, and 4-day periods in preschool children’s FMS, which warrants further consideration, especially as FMS are referred to as a prerequisite for PA and children’s future health [15,57,63,64].

Surprisingly, increasing SB at the expense of LPA in the weekend-derived behaviours only was associated with positive changes in all outcome variables, whilst adding SB at the expense of MVPA in the week-derived behaviours was associated with a small, but significant, improvement in object control and motor competence, but not in locomotor skills; this differs from the results of Roscoe et al. [57] and Webster et al. [17], who did not observe any association between PA and FMS competency. Therefore, not only is the activity composition different between week, weekend and 4-day behaviours, but also the associated theoretical change in motor competence from reallocating 5 min of time indicates markedly different results. This would support work by Stodden et al. [64] indicating that greater exposure and increases in PA intensities allow for new motor experiences during the preschool years. This is not only increasing preschool children’s PA engagement in line with recommend guidelines, but ensuring greater health benefits [2]_._ The preschool years are a critical time to develop basic FMS [64]^,^ and higher motor competency allows for greater PA levels in adulthood [65]. Interestingly, differences were seen when data were considered compositionally (SB, LPA, MVPA) and adjusted for age, BMI and sex. In fact, the weekday-derived composition significantly predicted total motor competence (r^2^ = 0.07), locomotor (r^2^ = 0.08) and object control skills (r^2^ = 0.09); the weekend-derived composition significantly predicted total motor competence (r^2^ = 0.03) and object control skills (r^2^ = 0.03), the 4-day-derived composition significantly predicted total motor competence (r^2^ = 0.07), locomotor (r^2^ = 0.07), and object control skills (r^2^ = 0.06) (all *p* < 0.05). Generally, the larger the r^2^ value, the greater the meaningfulness of the correlation [66]. These key findings highlight discrepancies, in that different compositions, when adjusted for age, BMI and sex, significantly predicted different FMS skills, an observation that deserved further research and consideration when designing interventions for future implementation. As mentioned, adding SB at the expense of LPA and MVPA yielded significant improvements in object control, which could be attributed to preschool children developing handwriting, drawing and general fine motor skills and to the fact that these sedentary activities share commonalities with object control skills such as throwing and striking a ball, as they incorporate interactions between psychomotor skills, involving the nervous and muscular systems [67,68,69]. Theoretical changes in SB in this current study did not negatively affect FMS competency, similarly to what was found by Smith et al. [58], therefore highlighting the need to better identify types of SB in preschool children during the week and weekend days [70]. Through the identification of types of tasks, we can determine which activities can benefit preschool children. This, along with the intensity of the PA they perform, can guide the design of interventions to improve concurrently the PA and FMS of preschool children and consequently help to guide future health policies for them.

### Strengths and Limitations

As previously mentioned, children unwilling to participate in the study were removed. This was primarily due to their un-cooperation caused by shyness; however, this may have introduced a selection bias, since some children may have been unwilling because they had low actual or perceived competence, which is likely to affect their physical activity and movement behaviours and subsequently the relationship between these two variables. The use of the CoDA approach was based on the validated and objective measurement of PA (GENEActiv accelerometer). In addition, the use of the TGMD-2 process-orientated assessment to measure the FMS of preschool children was important, as it is validated for this age range. Indeed, both measurements (PA and FMS) were used to reduce any potential bias or measurement error and allow comparisons across the literature. That said, accelerometers cannot completely distinguish between sitting and standing postures, with both behaviours potentially being classified as sedentary [71]; therefore, this could be viewed as something which warrants further research, particularly given the interesting finding that increasing SB was in part positively associated with improvements in object control. It should be noted that the authors hypothesised that improvements in FMS when adding SB and removing LPA/MVPA could be a result of preschool children working on their fine motor skills when participating in SB, as we only recorded activity counts and not contextual information. However, this would be an area to further investigate. This study was performed in an area of low-socioeconomic status, which in England is linked to lower PA levels [72], which allowed for the data to be representative of this population; clearly, however, assessing children from different SES backgrounds is suggestible. The preschool children in this study were predominately Caucasian; therefore, children of all ethnicities need to be assessed to ensure comparisons can be made with the general population.

## 5. Conclusions

To our knowledge, this is the first study to address how week, weekend, and four-day movement compositions, separately, associate with FMS. The results highlight that adding LPA and removing SB was associated with improvements in the children’s total motor competence, locomotor and object control skills, when considering week, weekend and 4-day movement compositions. Interestingly, increasing SB at the expense of LPA in the weekend behaviours was associated with positive changes in all outcome variables, whilst adding SB at the expense of MVPA in the week behaviours was associated with a small, but significant, improvement in object control but not in locomotor skills. Not only is the composition different between week, weekend and 4-day behaviours, but the associated theoretical change in FMS from reallocating 5 min of time indicates markedly different results. Further investigation of movement behaviours over different time periods is advocated to better understand the impact that PA compositions have on locomotor, object control and total FMS in preschool children.

## Figures and Tables

**Table 1:** Samples’ descriptive statistics.

	Week	Weekend	4-Day
SB (min·day^−1^)	559.72	586.80	570.99
SB (comp)	0.93	0.98	0.95
LPA (min·day^−1^)	6.57	4.60	5.78
LPA (comp)	0.01	0.01	0.01
MVPA (min·day^−1^)	33.71	8.60	23.23
MVPA (comp)	0.06	0.01	0.04

Note: SB: sedentary behaviour; LPA: light physical activity; MVPA: moderate-to-vigorous physical activity.

**Table 2:** Behaviour variation matrix.

	SB			LPA			MVPA		
	WEEK	WKD	4	WEEK	WKD	4	WEEK	WKD	4
SB	-	-	-	0.36	0.49	030	0.22	0.93	0.23
LPA	0.36	0.49	0.30	-	-	-	0.52	0.99	0.53
MVPA	0.22	0.93	0.23	0.52	0.99	0.53	-	-	-

SB: sedentary time; LPA: light physical activity; MVPA: moderate-to-vigorous physical activity; WEEK: week-derived behaviours; WKD: weekend-derived behaviours; 4: 4-day-derived behaviours.

**Table 3:** Linear regression of week, weekend and 4-day behaviours.

	Sedentary B [95% CI]			*p* Value (r^2^)			LPA B [95% CI]			*p* Value (r^2^)			MVPA B [95% CI]			*p* Value (r^2^)		
	WEEK	WKD	4-day	WEEK	WKD	4-day	WEEK	WKD	4-day	WEEK	WKD	4-day	WEEK	WKD	4-day	WEEK	WKD	4-day
LOCO	−0.01 [−0.15, 0.13]	−0.03 [−0.18, 0.11]	−0.04 [−0.19, 0.10]	0.89 (0.001)	0.65 (0.001)	0.65 (0.001)	0.25 [0.22, 0.39]	0.18 [0.03, 0.32]	0.24 [0.10, 0.39]	0.007 * (0.06)	0.01 * (0.03)	0.008 * (0.06)	−0.008 [−0.15, 0.14]	0.06 [−0.08, 0.21]	0.01 [−0.13, 0.16]	0.91 (0.0007)	0.41 (0.03)	0.81 (0.0003)
OBJ.	0.08 [−0.06, 0.28]	0.12 [−0.02, 0.27]	0.13 [−0.01, 0.28]	0.25 (0.007)	0.1 (0.02)	0.07 (0.02)	0.21 [0.06, 0.35]	0.20 [0.05, 0.34]	0.21 [0.08, 0.36]	0.005 * (0.04)	0.006 * (0.04)	0.003 * (0.05)	−0.04 [−0.19, 0.10]	0.04 [−0.10, 0.19]	−0.03 [−0.17, 0.11]	0.53 (0.002)	0.54 (0.002)	0.68 (0.0009)
Total MC	0.02 [−0.12, 0.17]	0.03 [−0.23, 0.18]	0.03 [−0.11, 0.18)	0.71 (0.007)	0.72 (0.008)	0.68 (0.009)	0.25 [0.11, 0.40]	0.21 [0.06, 0.35]	0.26 [0.12, 0.41]	0.006 * (0.07)	0.005 * (0.04)	0.005 * (0.06)	−0.02 [−0.17, 0.12]	0.06 [−0.08, 0.20]	0.00 [−0.14, 0.14]	0.74 (0.006)	0.41 (0.003)	0.99 (0.00007)

Note. All adjusted for age, BMI and sex. B: Beta coefficient; CI: confidence interval; LPA: light physical activity; MVPA: moderate-to-vigorous physical activity; WEEK: week-derived behaviours; WKD: weekend-derived behaviours; 4: 4-day-derived behaviours; LOCO: locomotor subset score; OBJ.: object control subset score; Total MC: total motor competence score; * significant at <0.05.

**Table 4:** Three-behavior isotemporal substitution.

		Add	SB			SB			LPA			LPA			MVPA			MVPA		
		Remove	LPA			MVPA			SB			MVPA			SB			LPA		
			WEEK	WKD	4	WEEK	WKD	4	WEEK	WKD	4	WEEK	WKD	4	WEEK	WKD	4	WEEK	WKD	4
TOTAL MC	Total (95% CI)	5 min	−6.18 * (−8.84, −3.52)	6.50 * (2.28, 10.72)	−10.07 * (−13.95, −6.19)	0.26 * (0.28, 0.81)	−0.23 (−1.43, 0.97)	0.01 (−0.81, 0.82)	2.45 * (1.39, 3.52)	1.77 * 0.63, 2.92)	3.16 * 1.94, 4.38)	2.72 * (1.51, 3.93)	1.54 (−0.37, 3.45)	3.16 * (1.71, 4.62)	−0.22 (0.70, 0.25)	0.13 (−0.50, 0.77)	0.00 (−0.66, 0.67)	−6.40 * (−9.13, −3.68)	7.17 * (2.77, 11.56)	−10.07 * (−14.00, −6.13)
LOCO		5 min	−3.69 * (−5.49, −1.88)	3.76 * (0.86, 6.65)	−6.54 * (−9.20, −3.88)	0.09 (−0.28, 0.46)	−0.22 (−1.04, 0.60)	−0.17 −0.73, 0.39)	1.47 * (0.75, 2.19)	1.03 * (0.24, 1.81)	2.05 * (1.22, 2.89)	1.558 (0.74, 2.37)	0.81 (−0.50, 2.12)	1.88 * (0.89, 2.88)	−0.07 (−0.39, 0.25)	0.12 (−0.31, 0.56)	0.14 (−0.31, 0.60)	−3.76 * (−5.61, −1.91)	4.19 * (1.17, 7.21)	−6.39 * −9.09, −3.70)
OBJ.		5 min	−2.49 * (−3.57, −1.42)	2.74 * (1.02, 4.47)	−3.53 * (−5.13, −1.94)	0.17 * (−0.04, 0.39)	−0.01 (−0.50, 0.48)	0.17 −0.16, 0.51)	0.99 * (0.56, 1.42)	0.75 * (0.28, 1.21)	1.10 * (0.60, 1.61)	1.16 * (0.67, 1.65)	0.73 (−0.05, 1.52)	1.28 * (0.68, 1.88)	−0.15 (−0.34, 0.04)	0.01 (−0.25, 0.27)	−0.14 (−0.41, 0.13)	−2.64 * (−3.74, −1.54)	2.98 * (1.18, 4.78)	−3.67 * (−5.29, −2.05)

Note: CI: confidence interval; SB: sedentary time; LPA: light physical activity; MVPA: moderate-to-vigorous physical activity; TOTAL MC: total motor competence score; LOCO: locomotor subset score; OBJ.: object control subset score; WEEK: week-derived behaviours; WKD: weekend-derived behaviours; 4: 4-day-derived behaviours; * significant at <0.05.

## Data Availability

The data presented in this study are available on request from the corresponding author. The data are not publicly available due to ethical and GDPR reasons.

## References

[1] Adolph A.L., Puyau M.R., Vohra F.A., Nicklas T.A., Zakeri I.F., Butte N.F. (2012). Validation of uniaxial and triaxial accelerometers for the assessment of physical activity in preschool children. J. Phys. Act. Health.

[2] Carson V., Lee E.-Y., Hewitt L., Jennings C., Hunter S., Kuzik N., Stearns J.A., Powley Unrau S., Poitras V.J., Gray C. (2017). Systematic review of the relationships between physical activity and health indicators in the early years (0–4 years). BMC Public Health.

[3] Esliger D.W., Tremblay M.S. (2007). Physical activity and inactivity profiling: The next generation. Appl. Physiol. Nutr. Metab..

[4] Tremblay M.S., Chaput J.-P., Adamo K.B., Aubert S., Barnes J.D., Choquette L., Duggan M., Faulkner G., Goldfield G.S., Gray C.E. (2017). Canadian 24-Hour Movement Guidelines for the Early Years (0–4 years): An Integration of Physical Activity, Sedentary Behaviour, and Sleep. BMC Public Health.

[5] UK Chief Medical Officers’ Physical Activity Guidelines. https://assets.publishing.service.gov.uk/government/uploads/system/uploads/attachment_data/file/832868/uk-chief-medical-officers-physical-activity-guidelines.pdf.

[6] Jackson D.M., Reilly J.J., Kelly L.A., Montgomery C., Grant S., Paton J.Y. (2003). Objectively measured physical activity in a representative sample of 3- to 4-year-old children. Obes. Res..

[7] Montgomery C., Reilly J.J., Jackson D.M., Kelly L.A., Slater C., Paton J.Y., Grant S. (2004). Relation between physical activity and energy expenditure in a representative sample of young children. Am. J. Clin. Nutr..

[8] Pate R.R., McIver K., Dowda M., Brown W.H., Addy C. (2008). Directly observed physical activity levels in preschool children. J. Sch. Health.

[9] Raustorp A., Pagels P., Boldemann C., Cosco N., Söderström M., Mårtensson F. (2012). Accelerometer measured level of physical activity indoors and outdoors during preschool time in Sweden and the United States. J. Phys. Act. Health.

[10] Reilly J.J. (2010). Low levels of objectively measured physical activity in preschoolers in child care. Med. Sci. Sports Exerc..

[11] Roscoe C.M.P., James R.S., Duncan M.J. (2019). Accelerometer-Based Physical Activity Levels Differ between Week and Weekend Days in British Preschool Children. J. Funct. Morphol. Kinesiol..

[12] The Physical Activity Levels of Preschool-Aged Children: A Systematic Review. https://www.sciencedirect.com/science/article/pii/S0885200608000719.

[13] Tinner L., Kipping R., White J., Jago R., Metcalfe C., Hollingworth W. (2019). Cross-sectional analysis of physical activity in 2-4-year-olds in England with paediatric quality of life and family expenditure on physical activity. BMC Public Health.

[14] Logan S.W., Ross S.M., Chee K., Stodden D.F., Robinson L.E. (2018). Fundamental motor skills: A systematic review of terminology. J. Sports Sci..

[15] Logan S.W., Robinson L.E., Wilson A.E., Lucas W.A. (2012). Getting the fundamentals of movement: A meta-analysis of the effectiveness of motor skill interventions in children. Child. Care Health Dev..

[16] van Beurden E., Zask A., Barnett L.M., Dietrich U.C. (2002). Fundamental movement skills--how do primary school children perform? The ‘Move it Groove it’ program in rural Australia. J. Sci. Med. Sport.

[17] Webster E.K., Martin C.K., Staiano A.E. (2019). Fundamental motor skills, screen-time, and physical activity in preschoolers. J. Sport Health Sci..

[18] Williams H.G., Pfeiffer K.A., O’Neill J.R., Dowda M., McIver K.L., Brown W.H., Pate R.R. (2008). Motor skill performance and physical activity in preschool children. Obes. Silver Spring Md..

[19] Hesketh K.R., McMinn A.M., Ekelund U., Sharp S.J., Collings P.J., Harvey N.C., Godfrey K.M., Inskip H.M., Cooper C., van Sluijs E.M.F. (2014). Objectively measured physical activity in four-year-old British children: A cross-sectional analysis of activity patterns segmented across the day. Int. J. Behav. Nutr. Phys. Act..

[20] Okely A.D., Booth M.L., Chey T. (2004). Relationships between body composition and fundamental movement skills among children and adolescents. Res. Q. Exerc. Sport.

[21] Hesketh K.R., Griffin S.J., van Sluijs E.M.F. (2015). UK Preschool-aged children’s physical activity levels in childcare and at home: A cross-sectional exploration. Int. J. Behav. Nutr. Phys. Act..

[22] Møller N.C., Christensen L.B., Mølgaard C., Ejlerskov K.T., Pfeiffer K.A., Michaelsen K.F. (2017). Descriptive analysis of preschool physical activity and sedentary behaviors—A cross sectional study of 3-year-olds nested in the SKOT cohort. BMC Public Health.

[23] O’Dwyer M., Fairclough S.J., Ridgers N.D., Knowles Z.R., Foweather L., Stratton G. (2014). Patterns of objectively measured moderate-to-vigorous physical activity in preschool children. J. Phys. Act. Health.

[24] Hinkley T., Salmon J., Okely A.D., Hesketh K., Crawford D. (2012). Correlates of preschool children’s physical activity. Am. J. Prev. Med..

[25] Berglind D., Tynelius P. (2017). Objectively measured physical activity patterns, sedentary time and parent-reported screen-time across the day in four-year-old Swedish children. BMC Public Health.

[26] Soini A., Villberg J., Sääkslahti A., Gubbels J., Mehtälä A., Kettunen T., Poskiparta M. (2014). Directly observed physical activity among 3-year-olds in Finnish childcare. Int. J. Early Child..

[27] Mota J.G., Clark C.C.T., Bezerra T.A., Lemos L., Reuter C.P., Mota J.A.P.S., Duncan M.J., Martins C.M. (2020). Twenty-four-hour movement behaviours and fundamental movement skills in preschool children: A compositional and isotemporal substitution analysis. J. Sports Sci..

[28] JSNA Place-Based Approach. https://www.warwickshire.gov.uk/joint-strategic-needs-assessments-1/jsna-place-based-approach.

[29] Trost S.G., Sirard J.R., Dowda M., Pfeiffer K.A., Pate R.R. (2003). Physical activity in overweight and nonoverweight preschool children. Int. J. Obes..

[30] Barlow S.E., Dietz W.H. (1998). Obesity evaluation and treatment: Expert Committee recommendations. The Maternal and Child Health Bureau, Health Resources and Services Administration and the Department of Health and Human Services. Pediatrics.

[31] Bellizzi M.C., Dietz W.H. (1999). Workshop on childhood obesity: Summary of the discussion. Am. J. Clin. Nutr..

[32] Dietz W.H., Bellizzi M.C. (1999). Introduction: The use of body mass index to assess obesity in children. Am. J. Clin. Nutr..

[33] Himes J.H., Dietz W.H. (1994). Guidelines for overweight in adolescent preventive services: Recommendations from an expert committee. The Expert Committee on Clinical Guidelines for Overweight in Adolescent Preventive Services. Am. J. Clin. Nutr..

[34] Lehto R., Ray C., Lahti-Koski M., Roos E. (2011). Health behaviors, waist circumference and waist-to-height ratio in children. Eur. J. Clin. Nutr..

[35] McCarthy H.D., Jarrett K.V., Crawley H.F. (2001). The development of waist circumference percentiles in British children aged 5.0-16.9 y. Eur. J. Clin. Nutr..

[36] Hasselstrøm H., Karlsson K.M., Hansen S.E., Grønfeldt V., Froberg K., Andersen L.B. (2007). Peripheral bone mineral density and different intensities of physical activity in children 6-8 years old: The Copenhagen School Child Intervention study. Calcif. Tissue Int..

[37] Obeid J., Nguyen T., Gabel L., Timmons B.W. (2011). Physical activity in Ontario preschoolers: Prevalence and measurement issues. Appl. Physiol. Nutr. Metab..

[38] Vale S., Santos R., Silva P., Soares-Miranda L., Mota J. (2009). Preschool children physical activity measurement: Importance of epoch length choice. Pediatr. Exerc. Sci..

[39] Choi L., Liu Z., Matthews C.E., Buchowski M.S. (2011). Validation of accelerometer wear and nonwear time classification algorithm. Med. Sci. Sports Exerc..

[40] Benham-Deal T. (2005). Preschool children’s accumulated and sustained physical activity. Percept. Mot. Skills..

[41] Roscoe C.M.P., James R.S., Duncan M.J. (2017). Calibration of GENEActiv accelerometer wrist cut-points for the assessment of physical activity intensity of preschool aged children. Eur. J. Pediatr..

[42] GENEActiv Support|Activinsights Professional Wearables & Research Accelerometers. https://www.activinsights.com/expertise/geneactiv/.

[43] Ulrich D.A., Sanford C.B. (2000). Test. of Gross Motor Development: Examiner’s Manual.

[44] Frost J.L., Wortham S.C., Reifel S.C. (2011). Play and Child Development.

[45] Sallis J.F., Prochaska J.J., Taylor W.C. (2000). A review of correlates of physical activity of children and adolescents. Med. Sci. Sports Exerc..

[46] Barnett L., Hinkley T., Okely A.D., Salmon J. (2013). Child, family and environmental correlates of children’s motor skill proficiency. J. Sci. Med. Sport.

[47] Hardy L.L., King L., Farrell L., Macniven R., Howlett S. (2010). Fundamental movement skills among Australian preschool children. J. Sci. Med. Sport.

[48] Ulrich D.A. (2019). Test. of Gross Motor Developmet-3 (TGMD-3): Examiners Manual.

[49] Foulkes J.D., Knowles Z., Fairclough S.J., Stratton G., O’Dwyer M., Ridgers N.D., Foweather L. (2015). Fundamental movement skills of preschool children in northwest england. Percept. Mot. Skills.

[50] van der Mars H. (1989). Chapter 3-Observer Reliability-Issues & Procedures.

[51] “Compositions”: A Unified R Package to Analyze Compositional Data. https://www.sciencedirect.com/science/article/pii/S009830040700101X.

[52] Robcompositions: An R-Package for Robust Statistical Analysis of Compositional Data-Compositional Data. https://onlinelibrary.wiley.com/doi/abs/10.1002/9781119976462.ch25.

[53] The Statistical Analysis of Compositional Data. https://www.jstor.org/stable/2345821.

[54] Chastin S.F.M., Palarea-Albaladejo J., Dontje M.L., Skelton D.A. (2015). Combined Effects of Time Spent in Physical Activity, Sedentary Behaviors and Sleep on Obesity and Cardio-Metabolic Health Markers: A Novel Compositional Data Analysis Approach. PLoS ONE.

[55] Fox J., Weisberg S. An R Companion to Applied Regression. https://us.sagepub.com/en-us/nam/an-r-companion-to-applied-regression/book246125.

[56] Foweather L., Knowles Z., Ridgers N.D., O’Dwyer M.V., Foulkes J.D., Stratton G. (2015). Fundamental movement skills in relation to weekday and weekend physical activity in preschool children. J. Sci. Med. Sport.

[57] Roscoe C.M.P., James R.S., Duncan M.J. (2019). Accelerometer-based physical activity levels, fundamental movement skills and weight status in British preschool children from a deprived area. Eur. J. Pediatr..

[58] Smith E., Fazeli F., Wilkinson K., Clark C.C.T. (2021). Physical behaviors and fundamental movement skills in British and Iranian children: An isotemporal substitution analysis. Scand. J. Med. Sci. Sports.

[59] Chen B., Bernard J.Y., Padmapriya N., Yao J., Goh C., Tan K.H., Yap F., Chong Y.-S., Shek L., Godfrey K.M. (2019). Socio-demographic and maternal predictors of adherence to 24-hour movement guidelines in Singaporean children. Int. J. Behav. Nutr. Phys. Act..

[60] Westerterp K.R., Plasqui G. (2004). Physical activity and human energy expenditure. Curr. Opin. Clin. Nutr. Metab. Care.

[61] Guidelines on Physical Activity, Sedentary Behaviour, and Sleep for Children under 5 Years of Age. http://www.ncbi.nlm.nih.gov/books/NBK541170/.

[62] To Grow Up Healthy, Children Need to Sit Less and Play More. https://www.who.int/news/item/24-04-2019-to-grow-up-healthy-children-need-to-sit-less-and-play-more.

[63] Bryant E.S., Duncan M.J., Birch S.L. (2014). Fundamental movement skills and weight status in British primary school children. Eur. J. Sport Sci..

[64] Stodden D.F., Goodway J.D., Langendorfer S.J., Roberton M.A., Rudisill M.E., Garcia C., Garcia L.E. (2008). A Developmental Perspective on the Role of Motor Skill Competence in Physical Activity: An Emergent Relationship. Quest.

[65] Smith L., Fisher A., Hamer M. (2015). Prospective association between objective measures of childhood motor coordination and sedentary behaviour in adolescence and adulthood. Int. J. Behav. Nutr. Phys. Act..

[66] Thomas J., Nelson J., Silverman S. (2015). Research Methods in Physical Activity.

[67] Accardo A.P., Genna M., Borean M. (2013). Development, maturation and learning influence on handwriting kinematics. Hum. Mov. Sci..

[68] Barr-Anderson D.J., Robinson-O’Brien R., Haines J., Hannan P., Neumark-Sztainer D. (2010). Parental report versus child perception of familial support: Which is more associated with child physical activity and television use?. J. Phys. Act. Health.

[69] Biddle S.J.H., Gorely T., Marshall S.J. (2009). Is television viewing a suitable marker of sedentary behavior in young people?. Ann. Behav. Med..

[70] Stamatakis E., Coombs N., Jago R., Gama A., Mourão I., Nogueira H., Rosado V., Padez C. (2013). Type-specific screen time associations with cardiovascular risk markers in children. Am. J. Prev. Med..

[71] Dumuid D., Stanford T.E., Pedišić Ž., Maher C., Lewis L.K., Martín-Fernández J.-A., Katzmarzyk P.T., Chaput J.-P., Fogelholm M., Standage M. (2018). Adiposity and the isotemporal substitution of physical activity, sedentary time and sleep among school-aged children: A compositional data analysis approach. BMC Public Health.

[72] Khashayar P., Kasaeian A., Heshmat R., Motlagh M.E., Mahdavi Gorabi A., Noroozi M., Qorbani M., Kelishadi R. (2018). Childhood Overweight and Obesity and Associated Factors in Iranian Children and Adolescents: A Multilevel Analysis; the CASPIAN-IV Study. Front. Pediatr..

